# Disposable Optical Stretcher Fabricated by Microinjection Moulding

**DOI:** 10.3390/mi9080388

**Published:** 2018-08-04

**Authors:** Gianluca Trotta, Rebeca Martínez Vázquez, Annalisa Volpe, Francesco Modica, Antonio Ancona, Irene Fassi, Roberto Osellame

**Affiliations:** 1 Institute of Intelligent Industrial Technologies and Systems for Advanced Manufacturing, National Research Council, 70124 Bari, Italy; Gianluca.Trotta@stiima.cnr.it (G.T.); Francesco.Modica@stiima.cnr.it (F.M.); 2 Institute for Photonics and Nanotechnologies, National Research Council, 20133 Milan, Italy; roberto.osellame@polimi.it; 3 Institute for Photonics and Nanotechnologies, National Research Council, 70126 Bari, Italy; annalisa.volpe@ifn.cnr.it (A.V.); antonio.ancona@ifn.cnr.it (A.A.); 4 Institute of Intelligent Industrial Technologies and Systems for Advanced Manufacturing, National Research Council, 20133 Milan, Italy; Irene.Fassi@stiima.cnr.it

**Keywords:** microfluidics, microinjection moulding, femtosecond laser micromachining, optical manipulation

## Abstract

Microinjection moulding combined with the use of removable inserts is one of the most promising manufacturing processes for microfluidic devices, such as lab-on-chip, that have the potential to revolutionize the healthcare and diagnosis systems. In this work, we have designed, fabricated and tested a compact and disposable plastic optical stretcher. To produce the mould inserts, two micro manufacturing technologies have been used. Micro electro discharge machining (µEDM) was used to reproduce the inverse of the capillary tube connection characterized by elevated aspect ratio. The high accuracy of femtosecond laser micromachining (FLM) was exploited to manufacture the insert with perfectly aligned microfluidic channels and fibre slots, facilitating the final composition of the optical manipulation device. The optical stretcher operation was tested using microbeads and red blood cells solutions. The prototype presented in this work demonstrates the feasibility of this approach, which should guarantee real mass production of ready-to-use lab-on-chip devices.

## 1. Introduction

The development of miniaturized lab-on-chip (LoC) devices, able to perform analysis on very small volumes of biological samples through optical or electrical probes, is a growing and interdisciplinary field that will help to improve the comprehension of basic biological mechanisms, above all when the analysis is performed at a single cell level [1,2]. In particular, several results reported in the scientific literature demonstrate that single cell mechanical properties are reliable markers of a specific cell status [3].

An optical stretcher represents an accurate, non-invasive and gentle manipulation technique to study mechanical properties of singles cells. It relies on the exploitation of optical forces to study the viscoelastic properties of individual cells; an individual cell (flowing inside a microfluidic channel) is trapped between two divergent, opposing laser beams. A stress is exerted on the cell where the light hits the surface causing an elongation of the cell body along the laser beam axis (stress-strain elasticity experiment) [4]. The development of disposable integrated stretcher devices for single cell analysis will pave the way for its massive exploitation in laboratories as a tool for early diagnosis of diseases [5].

Traditional approaches to optical stretcher fabrication rely on the use of conventional inorganic substrates such as glass [6,7]. If looking for practical low-cost and disposable devices, it would be preferable to replace these materials with polymeric substrates like elastomers or thermoplastics that are already broadly used in LoC manufacturing. In particular, polydimethylsiloxane (PDMS) is a transparent elastomer that is cheap and easy to manipulate. It is largely used in laboratory applications for the prototyping and testing of LoC devices. Nevertheless, PDMS presents some limitations for industrial applications: it’s mechanical properties depend on the initial mixture, many surface structures are not permanent due to its relatively high chain mobility, under high fluid pressures the microchannels dimensions are not preserved and it is subject to aging [8,9,10]. An alternative solution for commercial applications is thermoplastic polymers, which have good mechanical and chemical properties for LoC applications, such as a high Young’s modulus, low water absorption, high solvent and acid/base resistance, and biocompatibility. Among thermoplastic polymers, cyclic olefin copolymer (COC), cyclic olefin polymer (COP) and poly(methyl methacrylate) (PMMA) are preferred for optical manipulation devices due to their high transparency in the visible spectrum [11,12]. However, it is important to consider that the quality of the moulded surface features is almost completely dependent on the quality and precision of the mould, and this will condition the transparency of the final device [12].

There are some examples in the literature of integrated optical stretchers fabricated with thermoplastic substrates; For example, Khoury Arvelo et al. [13] introduced a low-cost, all-polymer microfluidic chip fabricated by hot-embossing, with integrated deep-ultraviolet induced waveguides, which serves as a platform for optical manipulation of microbeads in a liquid flow. De Coster et al. [14] proposed a PMMA device in which fibre-optic laser beams are used to optically trap particles in a microchannel. The robust chip, made of PMMA, is obtained by double-sided hot embossing. Matteucci et al. [15] presented a chip produced in hard COC (TOPAS 5013) by injection moulding based on a nickel shim with a multi-layer structure used as experimental setups for optical stretching. However, there is still missing a broad use of these materials in research laboratory environments mainly because the fabrication of polymeric devices is more difficult and expensive to implement than PDMS.

There are different mass production methods to manufacture microfluidic devices made of thermoplastic materials, such as microinjection moulding (µIM), hot embossing or pressure injection [10]. Among them, µIM gives the highest throughput and is becoming a promising technology for the production of such devices thus attracting great interest from research, as evidenced by the numerous reviews published [11,16,17].

The weak point for a broad use of µIM is the requirement of moulds and tools that are relatively expensive and that limit the reconfigurability of the device layout. The current trend is toward parallelization of several functionalities in a single device, requiring a technological upgrade of the microinjection moulding process in order to increase its flexibility and to allow improvements of ongoing features, while maintaining a high level of replicability of the devices themselves.

In this direction, the mould becomes a micro-manufacturing laboratory in which different design strategies and micro-manufacturing techniques are experimented with to achieve the required level of precision and versatility.

In this work, we use a novel variation of microinjection moulding of PMMA, introducing the concept of removable and tailored inserts, structured by high-resolution femtosecond laser micromachining (FLM) and micro electro discharge machining (µEDM) [18]. FLM is able to process, with high removal rates, large surface areas with relatively small depth features, providing precision and surface roughness, and satisfying the specific requirements of the microfluidic application [19]. µEDM, by contrast, is the leading technology for high aspect ratio geometries due to the small removal rate, together with a fine pulse discharge that also ensures high precision and good surface roughness on the walls [20]. PMMA has been selected, among other thermoplastic polymers, because it is one of the cheapest, it has a high Young’s modulus and it can reproduce aspect ratios as low as 20 and a maximum structural thickness of 20 µm when moulded [11]. The obtained PMMA slabs were subsequently bonded with the femtosecond laser [21], avoiding problems related to thermal or glue bonding. The flexibility of this approach allows us to change the scheme of the microfluidic chip quickly and cost effectively compared with traditional µIM.

To prove its feasibility, we present, and validate, a single cell manipulator (an optical stretcher) which was seen to be an excellent example of a disposable device because it is cheap and easy to fabricate and to align. We demonstrate its working principle by trapping and stretching red blood cells (RBC). 

## 2. Device and µIM Mould Design

### 2.1. Optical Stretcher Design

**Figure 1:**
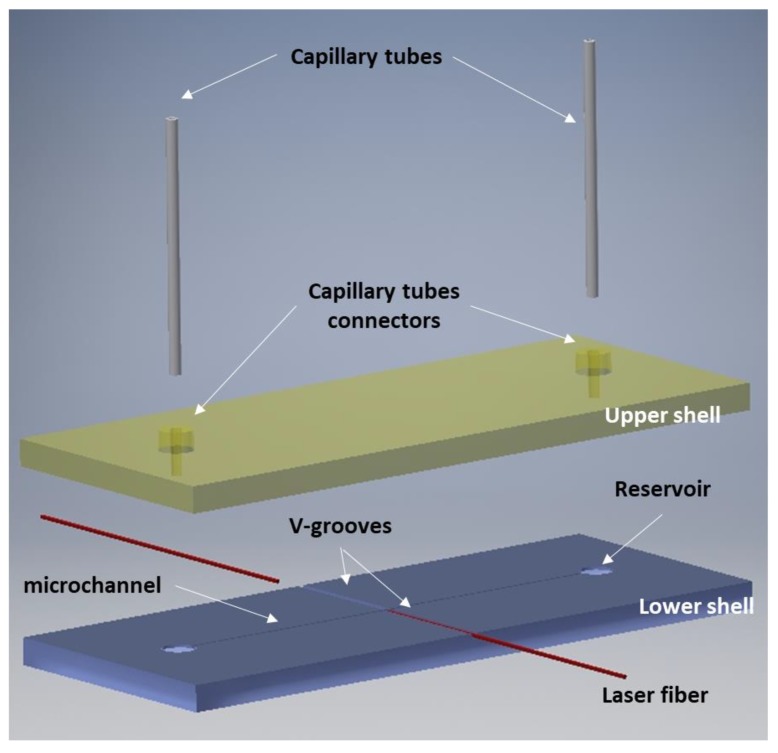
Design of the optical stretcher.

Figure 1 shows the design of the optical stretcher defined, taking under consideration the monolithic previous version reported in [7]. The device is composed of two shells, both of 19.5 mm length, 7.5 mm width and 0.7 mm thickness. The lower shell contains the main microfluidic features: a microchannel of 100 µm depth and 90 µm width, reservoirs of 1 mm diameter and V-grooves for laser fibre housing with 180 µm depth and 235 µm width. The upper shell closes the microfluidic chip and works as the interface with the external peristaltic pressure pump, through two capillary tubes connected to the capillary tubes housing. With respect to the original design, where the coaxial connectors were glued at the top of the upper shell, the updated design foresees a direct fabrication of the connectors on the upper shell (Figure 1), thus reducing the number of external components involved in the assembly. Moreover, a smoothest area in the center was realized in order to allow good imaging of the specimens flowing inside the microchannel. The shells were bonded together using the femtosecond laser welding approach reported in [21]. Here, after suitable clamping of the shells, non-linear absorption of the 1030-nm-wavelength femtosecond laser pulses focused at the interface, and heat accumulation generated by the high pulse spatial overlap caused by relatively slow beam movement and extremely high pulse repetition rate, caused localized melting and bonding with minimum collateral thermal damage to the surroundings. 

According to the analysis made in the introduction, the polymer PMMA (Acryrex^®^ CM-211 PMMA, Chi Mei Corp., Tainan City, Taiwan) was selected as the material for the device fabrication. 

### 2.2. Microinjection Moulding

The numerical simulation of microinjection moulding processes with Autodesk Moldflow Insight^®^ (2018, San Rafael, CA, USA) was used to define the final device design and to identify the optimal runner and gate design in order to avoid incomplete filling of the shell, while also facilitating the separation from the final component without damaging it.

**Figure 2:**
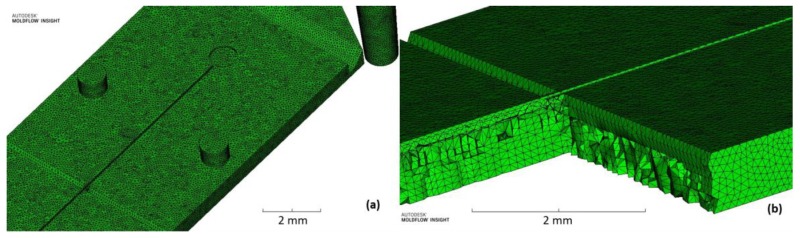
(**a**) Overview of mesh size (fine mesh at 100 µm with merge tolerance 10 µm) and (**b**) 3D mesh with number of element through thickness (10 elements).

A 3D fine mesh (Figure 2a,b) was used to reach sufficient contour accuracy (about 10 tetrahedra per micron) [22]. The bias ratio through thickness was not set as suggested in [23] because the thin plate proposed in this work has no variation in thickness geometry and this condition is helpful to reduce computation time.

**Figure 3:**
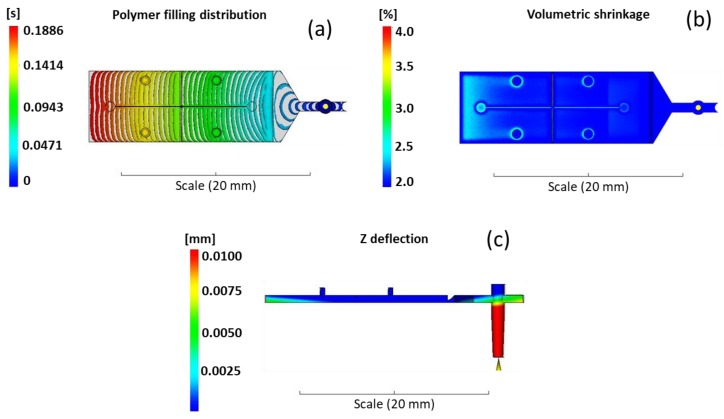
(**a**) Polymer filling distribution (time contour plot), (**b**) volumetric shrinkage (range 2%–4%), and (**c**) component deflection in the Z direction (out of XY plane).

The identification of the best process parameters followed the methodology introduced in Trotta et al. [24] and already tested by Martínez Vázquez et al. [18]. The filling behavior was analyzed in order to verify that the complementary geometries of the plastic part (sprue, runner and gate) do not interfere with the filling of the fluid by creating areas of hesitation or flow overlap. As shown in Figure 3a, the melt flow accelerates along the fan-gate, as expected, and then is uniformly distributed along the whole component without hesitation effects. A second parameter analyzed was the volumetric shrinkage. It allows estimation of the tension distribution in the material so that the warpage effect is reduced together with the deflection in plane of the part [22]. As shown in Figure 3b, the average volumetric shrinkage is between 2–4%, as suggested by the material datasheet, and the evaluation of the thin plate deflection (Figure 3c) in the Z direction (out of XY plane) is a few microns and localized in the last part of the component, where the volumetric shrinkage is higher than in the rest of the component. 

A set of process parameters that meet the criteria described above was identified with simulations and are reported in Table 1: melt temperature (T_melt_), mould temperature (T_mould_), injection speed (V_inj_), packing pressure (P_hold_), packing time (t_hold_), cooling time (t_cool_) and plunger injection run (Run).

**Figure 4:**
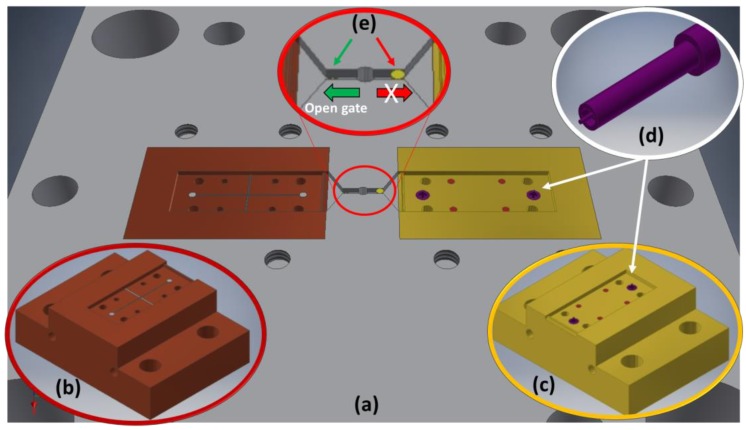
Design of the (**a**) mould plate with runners and gates, (**b**,**c**) main features inserts, (**d**) small inserts for capillary tube connectors and (**e**) overview of the interchangeable pins to alternatively close a runner while feeding another.

The design of the mould and of the tailored inserts (Figure 4a) aimed to satisfy the request of flexibility necessary to target the desired functionalities. The mould was conceived as an assembly of interchangeable inserts, each one used for a functionality [18]. The two cavities related to the two shells were designed symmetrically on two identical and interchangeable inserts (Figure 4b,c). Within the insert, smaller inserts were placed to reproduce, for example, the inverse of the housing for the capillary tubes (Figure 4d).

This design methodology has added considerable versatility to the mould because each micro feature can be easily replaced independently of the others, simply by replacing the related insert with the new one (Figure 4b,c). Furthermore, the strategy to realize a double symmetric gate, together with a single sprue on the same plate facing the inserts with cavities, was adopted (Figure 4a,e). In this way, the cavities are independent from the feeding system and therefore easily changeable. A further element that guarantees greater flexibility of the process in terms of maximum injectable volume usable for each cavity, as seen in [18], was also adopted in this case, and consists of several interchangeable pins having different heights. These can be used alternatively to close a runner while the other is open (Figure 4e) so that one cavity at a time can be filled by the polymer melt.

## 3. Fabrication

### 3.1. Inserts Manufacturing

**Figure 5:**
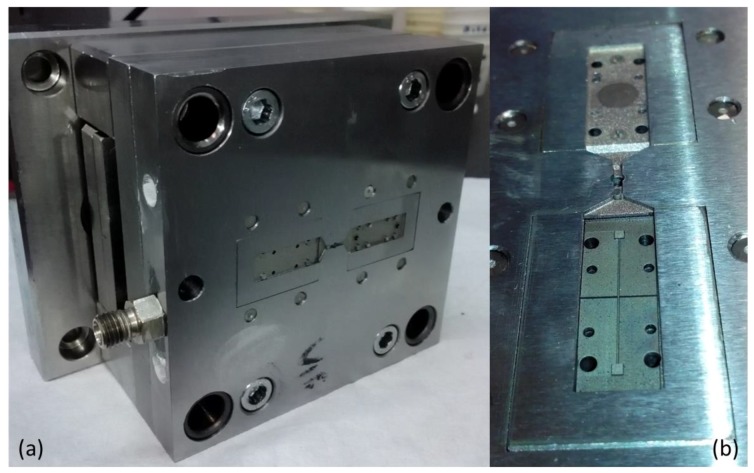
(**a**) Overview of the master mould and (**b**) of the inserts machined with femtosecond laser micromachining (FLM) and micro elactro discharge machining (µEDM) technologies.

The master mould and “bulk” inserts were fabricated with conventional manufacturing technologies such as milling and die sinking (Figure 5a) and then assembled in order to test the tolerances. Then the inserts (Figure 5b) were manufactured following two different strategies: the insert with microfluidic features (microchannel, reservoirs and V-grooves) was fabricated with FLM, while the insert with the inverse of the upper shell, with the smoothest area in the center and the inverse of the capillary tube housing inserts, was manufactured by µEDM technology.

#### 3.1.1. FLM

For FLM, we used an ultrafast fibre laser amplifier from Active Fiber Systems GmbH (Jena, Germany) based on the chirped pulse amplification technique (CPA). The laser source delivered an almost diffraction-limited beam (M^2^~1.25) at a wavelength of 1030 nm with a pulse duration in the range of 650 fs to 20 ps, a repetition rate varying from 50 kHz to 20 MHz, a maximum average power of 50 W and a maximum pulse energy of 100 μJ.

The linearly polarized beam exiting from the laser source was firstly converted into circularly polarized light by a quarter-wave-plate, and then focused and moved onto the target surface through a galvo-scan head (model Hurryscan from SCANLAB GmbH, Puchheim, Germany) equipped with a F-theta lens of 100 mm focal length. The resulting beam spot size on the metal surface was about 20 μm in diameter. The fs-laser milling process was carried out by removing the material layer by layer, by alternating overlapped vertical and horizontal hatch patterns of the laser beam onto the target surface. The ablation depth of the micro-milled area was finely controlled with micrometer precision by adjusting the number of scanning loops without affecting the surface quality.

After the milling process, the specimens were ultrasonically cleaned with isopropanol for 10 min to remove the redeposited debris. The ablation depth and the feature dimensions were measured by a confocal microscope (Zeiss Axio CSM 700, Carl Zeiss, Oberkochen, Germany) using a 10X lens. The roughness measurement was realized with the confocal microscopy using a 100X lens for a sample length of 4 mm. The calculation of the roughness is based on the DIN EN ISO 4287 standard.

**Figure 6:**
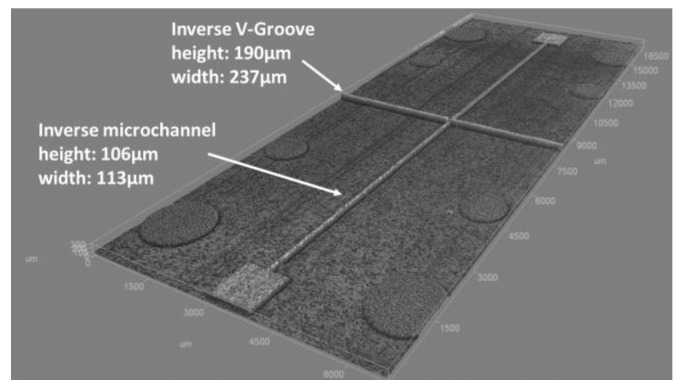
Machined “inverse” microchannel and V-grooves main dimensions measured with confocal microscope Zeiss Axio CSM 700 (magnification 10X with Numerical Aperture 0.25, lateral digital resolution of 160 nm and vertical resolution of 0.5 µm).

The best measured average surface roughness (Ra) was 59 nm, which was obtained at a laser repetition rate of 50 kHz, with a power of 400 mW and 7.05 TW/cm^2^ peak intensity at the focus of the F-theta lens, and moving the beam at a speed of 600 mm/s with a separation between adjacent scanned lines of 2 μm. Setting these parameters, the main dimensions of the features obtained are reported in Figure 6 and are in line with the expected values reported in Section 2.1.

#### 3.1.2. µEDM

**Figure 7:**
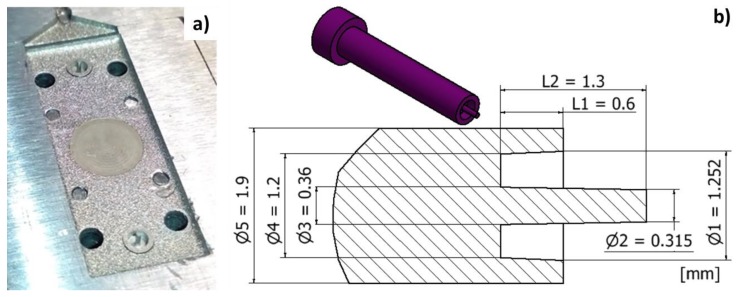
(**a**) Overview of the µEDM inserts with the capillary tube housing inserts and central circle machined with low roughness and (**b**) main dimensions of the capillary tube housing insert.

On the upper shell insert of Figure 7a are visible the small inserts for creating the capillary tube connectors. Main feature dimensions are illustrated in Figure 7b and are indicative of the manufacturing complexity due to the micro size, the thin central pillar and conical shapes. Due to low energy discharge and high accuracy, the µEDM process was adopted for inserts fabrication.

Starting from a cylindrical workpiece, assembled and referred to the main mould, the central conical pin was obtained by eroding the cavities following two operations. In the first operation, an electrode tool made of tungsten carbide (WC-Co) and having a diameter of 0.4 mm was adopted for reducing the cylindrical diameter and obtaining the first 0.7mm of the conical pin. In order to reduce the machining time, the energy level used was that for a semi-roughing regime. Since μEDM milling was implemented in a layer-by-layer strategy, a layer thickness of 0.004 mm was chosen to obtain a smooth surface and to avoid step-like effects. The machining time for this first operation was 45 min corresponding to a material removal rate (MRR) of 0.064 mm^3^/min. The second operation required the use of a smaller WC electrode tool, having a diameter of 0.15 mm, in order to machine the narrow cavity floor at the pin root. In order to prevent tool breakage (due to its small size), the spark energy level used in this machining step was that of a finishing regime with a layer thickness of 0.0012 mm. This operation started by eroding the cavity, beginning from a depth of 0.7 mm, and proceeding until the final conical pin profile was completed at a depth of 1.3 mm. This second operation was concluded in 11 h and 5 min, corresponding to an MRR of 0.001 mm^3^/min. It must be underlined that because the two operations were performed using two different machining regimes, different surface qualities were obtained: Ra = 0.9 µm for the semi-roughing regime and Ra = 0.6 µm for the finishing regime. The difference between these two surface quality values has been evaluated as acceptable.

#### 3.1.3. µIM Shells Manufacturing

The microinjection moulding machine used for the part manufacturing was the DESMA Tec Formica Plast 1K (DESMA Tec, Achim, Germany), a machine properly developed for microinjection moulding, characterized by a very small plunger (3 mm^2^), a maximum injection volume of 150 mm^3^ and an injection speed up to 500 mm/s.

The set of process parameters identified with simulation, and reported in Table 1, was used to produce the two shells of the optical stretcher without significant adjustments, as already highlighted in [24].

**Figure 8:**
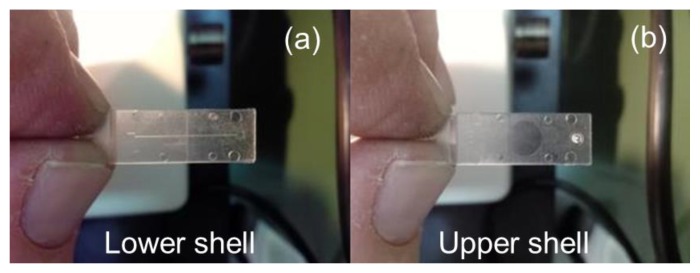
PMMA lower (**a**) and upper shells (**b**) of the Lab-on-chip prototype.

In Figure 8a,b the PMMA lower shell, with the microchannel and the V-grooves, and the PMMA upper shell, with the capillary tube connectors and smooth circle surface, are visible.

The measurements done with the confocal microscope (for which technical specifications are reported in Figure 6) are reported in Table 2. In particular, we compared the measurements of the lower shell microfeatures (Figure 8a) with the measurements of the related mould insert with the inverse of the microfeatures (Figure 6).

We focus on the comparison between the microchannel (µchannel) height (depth) and width, and V-groove height (depth) and width. The measures done on the mould insert were replicated three times while the measures done on the lower shell microfeatures were replicated three times, and each time on three different parts.

Analyzing the results, we can see that due to a large standard deviation of repeated measures of three different components microfeatures, the values µchannel width, µchannel height (depth) and V-groove height (depth) are in the range of the equivalent inverse feature measures on the mould insert. The most critical dimension is the V-groove width that has a minimal deviation of 2.8 µm from the inverse insert microfeature and a maximum deviation of 7.2 µm.

## 4. Optical Stretcher Demonstration

**Figure 9:**
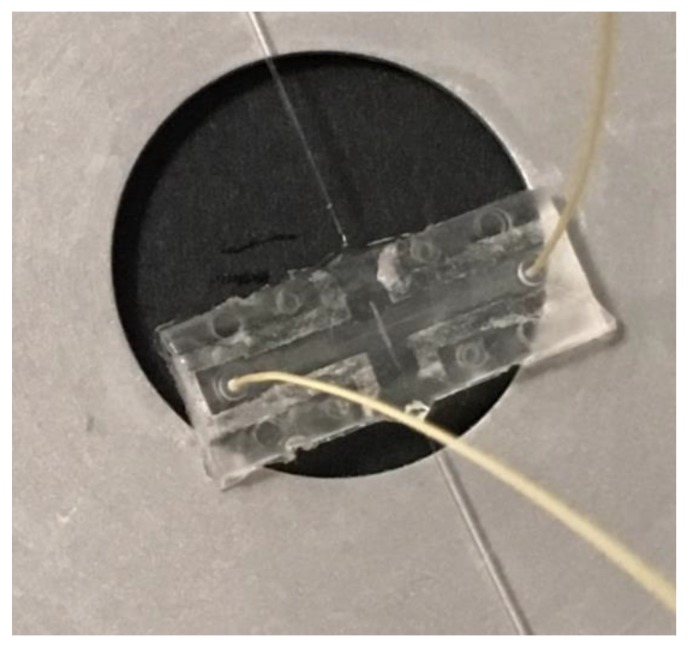
Picture of the final plastic optical stretcher with the PEEK tubes (yellow) and optical fibre inserted in it.

The final plastic optical stretcher consisted of a central channel and two closing ducts holding the optical fibres. No separation wall was present between the ducts and the microfluidic channel in order to avoid it being damaged by infrared light absorption during the trapping and stretching experiments. For the laser bonding of the two shells, we used the same femtosecond laser source utilized for the FLM but operated with a much higher repetition rate of 5 MHz and a pulse energy of 0.4 μJ. The two samples were previously mechanically clamped together and placed on an XY motorized translation table (Pro165LM, Aerotech, Inc., Pittsburgh, PA, USA) with micrometre resolution. The beam was focused at the interface between the two plates through a 0.3 numerical aperture lens. The welding path surrounded the central channel and the two V-groves (Figure 9) at a suitable distance to prevent thermal damage to the micro-features but, at the same time, ensuring sealing of the channel. The latter was proved by injecting a coloured liquid into the channel and verifying that there was no side leakage up to 1 bar of liquid pressure [21].

The fibre ducts were mutually aligned in order to ensure that the two counter-propagating beams were perfectly super positioned at a 20 µm depth with respect to the channel floor. In this position, the optical trap intercepted the maximum number of particles. A picture of the final device is shown in Figure 9, with the PEEK tubes (Outer diameter 360 µm, Upchurch Scientific^®^ PEEK™, IDEX Health & Science LLC, Oak Harbor, WA, USA) and the optical fibres inserted in their respective positions. The trace of the laser bonding around the microfluidic channel and ducts is clearly visible. In order to have a perfectly closed, robust and portable device once the fibres were inserted in their ducts, an active and fine alignment was done to ensure that the trapping efficiency is sufficient, and then they were glued with a UV curing glue (Norland Products Inc., Cranbury, NJ, USA).

**Figure 10:**
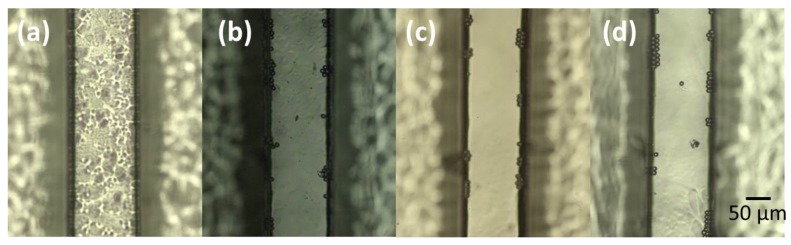
Microscope images of PMMA microinjected channels with no chloroform treatments (**a**) and with exposures times of (**b**) 2 minutes, (**c**) 4 minutes and (**d**) 6 minutes.

A preliminary inspection of the device under the microscope evidenced a channel roughness too high to avoid good phase contrast imaging. A chloroform vapour treatment was used in order to improve the transparency of the channel following the procedure reported by DeMarco et al. [25]. Figure 10 shows the microscope images of channels treated with different chloroform vapour exposures times. The flowing polystyrene beads allowed the better capture of the improvement in the imaging inside the channel from the pristine channel (a) to the channel treated for 4 minutes (c). It is worth noting that an overexposure (6 min) started to damage the channel surface.

For the device demonstration, a traditional optical stretcher setup was used. The laser source was a continuous wave ytterbium fiber laser (YLD-5k-1070, IPG Fibertech, Srl, Legnano, Mi, Italy) emitting up to 5 mW at 1070 nm. The output beam was split into two single mode fibres (HI-1060-Corning Incorporated, Corning, NY, USA) by a 50%-50% fibre coupler. The chip was mounted on to a phase contrast inverted microscope (Leica, Wetzlar, Germany) to capture the particles flowing, trapped and stretched in the microfluidic channel. The phase contrast images were acquired by a Charge-Coupled Device camera (DFC310 FX, Leica, Wetzlar, Germany) through a bandpass optical filter that cuts the infrared (IR) light (FGS550, Thorlabs, Newton, NJ, USA) [6].

**Figure 11:**
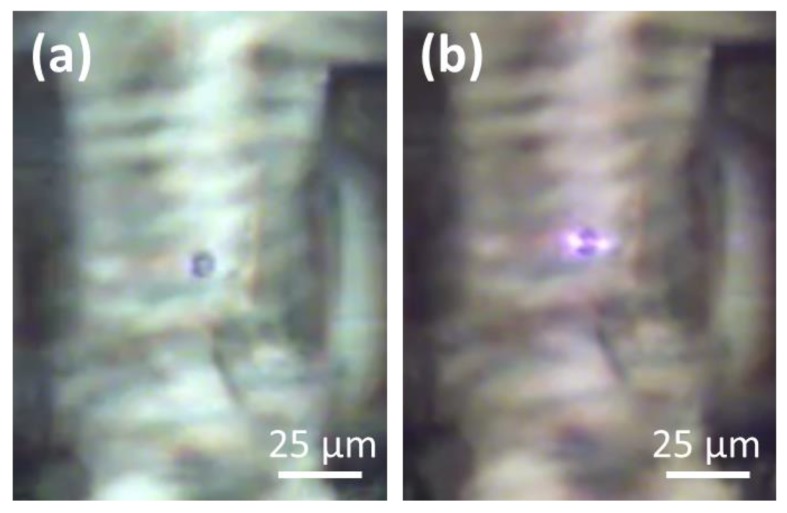
Image of a trapped polystyrene bead (7µm diameter) with the microscope infrared light (IR) light filter inside (**a**) or out (**b**). In (**b**) the scattering visible on both sides of the bead demonstrates the trapping due to the light from the optical fibres.

A preliminary test was done by inserting a solution of polystyrene microbeads (7 µm diameter) inside the microfluidic channel. It was possible to trap the micro-beads with less than 7 µW laser power on each branch. Figure 11 shows the phase contrast microscope images of a trapped bead with the IR light filter inserted (a) or not (b), in order to demonstrate that the bead was trapped due to the light beams out coming from the crossing optical fibres, impinging onto the bead surface from both sides.

The final trapping and stretching test was done using red blood cells (RBC). In order to obtain a sample of quasi-spherical RBCs with 8 µm diameter, we diluted 10 µL of blood in 8 mL of hypotonic solution. With a quasi-spherical cell, it will be easy to demonstrate stretching, as the cell profile will change from circular to elliptical when it will be elongated in the light propagation direction.

**Figure 12:**
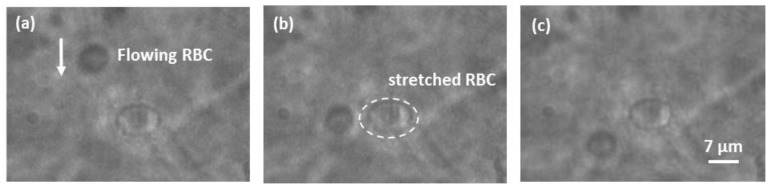
Sequence of frames of a stretched RBC (clearly elliptical). In the background is a non-trapped RBC (the arrow shows the flow direction). The non-trapped cell presents the typical circular profile of RBC in a hypotonic solution.

At flowing speeds in the range of 10 to 50 µL/s, RBC trapping was achieved with an estimated optical power coming out from each fiber of 30 mW. Once a single cell was trapped, it could be stretched by increasing the optical power coming out from the fibres, as shown in Figure 12. The stretched RBC shown was suffering a 40% elongation of its diameter with a laser power of 90 mW coming out from each fibre. The same image also shows a non-trapped RBC flowing inside the channel.

## 5. Conclusions

We have reported the design and manufacturing improvements of a PMMA lab-on-chip for optical manipulation of single cells produced with μIM technology using the interchangeable inserts approach.

The numerical simulation was used to assess the feasibility of improvements introduced in the original design, such as capillary tube connectors directly integrated in the upper shell, to identify criticalities, such as the centering pins that showed difficulty in ejecting the part, and to define the appropriate geometry of the cavity, runners and gate.

A modular design of the mould was conceived to exploit the flexibility achievable by the use of replaceable inserts, on which micro features were fabricated by fs-laser milling and μEDM machining. This choice improved the versatility of the manufacturing technique, allowing real mass production of ready-to-use micro devices.

The assembly of the final LoC was direct and easy, after the laser bonding of the two slabs. The capillary tubes and optical fibers were inserted into their dedicated housing; in particular, the optical fibres were well aligned when inserted in the fibre ducts. 

A validation of the stretching capabilities of the LoC with RBC solutions was undertaken, paving the way to a family of cheap and disposable single cell stretchers in the future.

## Figures and Tables

**Table 1:** Values for the microinjection moulding process parameters obtained from the simulations for Acryrex^®^ CM-211 PMMA (polymethylmethacrylate).

Material	T_melt_ (°C)	T_mould_ (°C)	V_inj_ (mm/s)	P_hold_ (bar)	t_hold_ (s)	t_cool_ (s)	Run (mm)
PMMA	250	80	100	1000	5	5	18

**Table 2:** Comparison between the measurements on the mould insert (Figure 6) and on the lower shell in PMMA.

Dimensions measured	Confocal measurement of the mould insert inverse microfeatures (Figure 6) (µm)	Confocal measurements of microfeatures on lower shell (µm)
µchannel width	113 ± 0.1	113 ± 2.8
µchannel height	106 ± 0.4	105 ± 1.2
V-groove height (depth)	190 ± 0.7	188 ± 1.2
V-groove width	237 ± 0.6	242 ± 1.6
